# ACHENY: A standard *Chenopodiaceae* image dataset for deep learning models

**DOI:** 10.1016/j.dib.2021.107478

**Published:** 2021-10-14

**Authors:** Ahmad Heidary-Sharifabad, Mohsen Sardari Zarchi, Sima Emadi, Gholamreza Zarei

**Affiliations:** a Department of Computer Engineering, Maybod Branch, Islamic Azad University, Maybod, Iran; b Department of Computer Engineering, Meybod University, Meybod, Iran; c Department of Computer Engineering, Yazd Branch, Islamic Azad University, Yazd, Iran; d Department of Agronomy, Maybod Branch, Islamic Azad University, Maybod, Iran

**Keywords:** Biodiversity protection, *Chenopodiaceae*, Deep learning, Image classification, Plant classification

## Abstract

This paper contains datasets related to the “Efficient Deep Learning Models for Categorizing *Chenopodiaceae* in the wild” (Heidary-Sharifabad et al., 2021). There are about 1500 species of *Chenopodiaceae* that are spread worldwide and often are ecologically important. Biodiversity conservation of these species is critical due to the destructive effects of human activities on them. For this purpose, identification and surveillance of *Chenopodiaceae* species in their natural habitat are necessary and can be facilitated by deep learning. The feasibility of applying deep learning algorithms to identify *Chenopodiaceae* species depends on access to the appropriate relevant dataset.

Therefore, ACHENY dataset was collected from natural habitats of different bushes of *Chenopodiaceae* species, in real-world conditions from desert and semi-desert areas of the Yazd province of IRAN. This imbalanced dataset is compiled of 27,030 RGB color images from 30 *Chenopodiaceae* species, each species 300-1461 images. Imaging is performed from multiple bushes for each species, with different camera-to-target distances, viewpoints, angles, and natural sunlight in November and December. The collected images are not pre-processed, only are resized to 224 × 224 dimensions which can be used on some of the successful deep learning models and then were grouped into their respective class. The images in each class are separated by 10% for testing, 18% for validation, and 72% for training. Test images are often manually selected from plant bushes different from the training set. Then training and validation images are randomly separated from the remaining images in each category. The small-sized images with 64 × 64 dimensions also are included in ACHENY which can be used on some other deep models.

## Specifications Table

**Table:** 

Subject	Computer Vision and Pattern Recognition, Deep Learning
Specific subject area	Image classification, Plant classification, *Chenopodiaceae* species identification
Type of data	Images in RGB color space (JPG).
How data were acquired	Imaging is performed using both Nikon COOLPIX S2800 digital camera with a 1: 1 (14.9-megapixel) 3864-by-3864 resolution and Samsung SM-J701F mobile with a 1: 1 (3.7-megapixel) 1920-by-1920 resolution.
Data format	Raw (unedited .JPG image files.) .jpeg
Parameters for data collection	Providing images of *Chenopodiaceae* species under unconstrained conditions in natural habitat of them.
Description of data collection	Imaging is performed on sunny, cloudy and windy days of November and December at different times in real-world conditions. Images are collected in the natural habitat of *Chenopodiaceae* species often from various visible organs of multiple bushes.
Data source location	Desert and semi-desert areas of the Yazd province of IRAN. Coordinates (longitude:52° 45′ .. 56° 30′, latitude:29° 48′ .. 33° 30′)
Data accessibility	Mendeley Data https://data.mendeley.com/datasets/fpfty8nn7j/1 doi:10.17632/fpfty8nn7j.1 Code reference doi:10.5281/zenodo.5151446
Related research article	Heidary-Sharifabad Ahmad,Sardari Zarchi Mohsen, Emadi Sima, Zarei Gholamreza, “Efficient Deep Learning Models for Categorizing *Chenopodiaceae* in the wild”, International Journal of Pattern Recognition and Artificial Intelligence, doi:10.1142/S0218001421520157

## Value of the Data

• This dataset is a resource for use by deep learning models and computer vision community and it can be used to advance plant classification researches.
• Automatic environment analysis, including tasks such as plant species recognition, *Chenopodiaceae* species identification in real-world, and imbalanced plant classification might benefit from this dataset.
• The ACHENY is a complex multiclass image dataset for researchers in the deep learning community for the development of image classification using computer vision methods.
• The first dataset for *Chenopodiaceae* species images in their natural habitat can be used to contribute biodiversity monitoring for their ecological impacts.
• This image dataset includes uncontrolled conditions with variations include viewpoint, intra-class, inter-class, rotation, illumination, and occlusion.
• ACHENY dataset can serve as a motivation to encourage further research into computer vision methods for plant species identification in the real-world. Researchers can use it during the development of new deep algorithms.

## 1 Data Description

*Chenopodiaceae* plants are mainly herbaceous and annual, but among them, there are also perennial, shrub and rarely tree or climber species. Stems and branches are often succulent, sometimes jointed. Leaves are alternate or opposite, exstipulate, herbaceous, succulent or reduced and scale-shaped. Flowers are small, bisexual or monosexual (monoecious or rarely dioecious), with uniseriate perianth or sometimes without perianth, placed in spike, panicle or cyme inflorescence. Perianths are actinomorphic often green, 4-5, rarely fewer, often enlarged and hardened in fruit, or winged. Stamens are 5 or fewer, ovary superior, 2–5 carpels, unilocular, and has 1 ovule. Fruits are achene, rarely pyxidium [2].

*Chenopodiaceae* species have spread throughout the world and have ecological and economic significance. In order to protect the biodiversity of *Chenopodiaceae* species, identifying them in their natural habitats is essential. Automatic plant identification can be performed using deep learning [3]. Applying deep learning techniques depends on the existence of a relevant dataset. Therefore, we collected the ACHENY (Autumn Chenopods of Yazd) dataset containing 27,030 images of 30 different *Chenopodiaceae* species. This image dataset was collected in real-world uncontrolled conditions using two usual imaging devices during November and December.

In Table 1 details of ACHENY were listed. The scientific name consists of a genus name and species name. To create class name the first 3 letters of species name were joined to the first 3 letters of the genus name. The class name and the natural habitat area of the studied species were also listed. The different numbers of images that were collected for each species were also listed in this table. 10% of collected images for each class were manually separated into test set that is often from distinct bushes from others, 72% randomly were separated to the training set, and the remaining 18% were assigned to the validation set. The images collected were not pre-processed, only they were resized to 224 × 224 dimensions and were placed in the appropriate folders and classes. A small version with 64 × 64 dimension images was also included in the ACHENY. Images with mentioned dimensions included in the ACHENY dataset are applicable to well-known deep models such as EfficientNet [4], VGG-16 [5], and MobileNet [6].

**Table 1:** ACHENY dataset details.

				Natural habitat (geographical location)			
	Scientific name				Coordinates		
Row	Genus	Species	Class name	Area name	Longitude	Latitude	Collected images #
**1**	Anabasis	haussknechtii Bge. Ex Boiss.	AnaHau	Around Nodooshan	53° 54′	32° 9′	769
**2**		setifera Moq.	AnaSet	Deserts areas around Yazd	54° 21′	31° 53′	1282
**3**	Atriplex	canescens (Pursh) Nutt.	AtrCan	Cultivated between Ardakan and Yazd	54° 0′	32° 18′	722
**4**		lentiformis (Torr.) S. Watds.	AtrLen	Cultivated in Chah-Afzal of Ardakan	53° 52′	32° 30′	1338
**5**		leucoclada (Boiss.) Aellen	AtrLeu	Desert areas around Yazd, Ardakan, and Maybod	54° 0′	32° 14′	629
**6**	Ceratocarpus	arenarius L.	CerAre	Around Taft (Shirkooh)	54° 13′	31° 46′	807
**7**	Chenopodium	album L.	CheAlb	Most areas of Yazd province among farms	54° 21′	31° 53′	652
**8**	Cornulaca	monacantha Delile	CorMon	Desert lands of Yazd: Between Yazd and Ardakan	53° 53′	32° 3′	1098
**9**	Girginsonia	oppositiflora (Pall.) Fenzl.	GirOpp	Around Mehriz	54° 26′	31° 34′	310
**10**	Haloxylon	ammodendron (C. A. Mey.) Bge.	HalAmm	Wide area of Yazd Deserts	54° 21′	31° 53′	929
**11**	Halostachys	belangeriana (Moq.) Botsch.	HalBel	Abarkuh deserts	53° 15′	31° 7′	628
**12**	Haloxylon	persicum Bge. Ex Boiss.	HalPer	From Mehriz to Marvast	54° 12′	30° 28′	1160
**13**	Halocnemum	strobiloceum (Pall.) M. B.	HalStr	Salt marshes around Behabad and Aghda	56° 1′	31° 52′	848
**14**	Halothamnus	subaphyllus (C. A. Mey.) Botsch.	HalSub	Around Chadormaloo	53° 43′	32° 23′	967
**15**	Hammada	salicornica (Moq.) lljin	HamSal	Yazd-Bafgh road	55° 24′	31° 37′	1191
**16**	Kochia	scoparia (L.) Schrad.	KocSco	Often as weed in parks	54° 4′	32° 13′	910
**17**		stellaris Moq.	KocSte	Yazd to Ardakan	54° 1′	32° 3′	851
**18**	Salsola	abarghuensis Assadi	SalAba	North of Abarkuh desert, around the village of Chah Beigi (endemic of Iran)	53° 15′	31° 23′	733
**19**		dendroides Pall.	SalDen	Between Marwast and Harat	54° 12′	30° 28′	1461
**20**		incanescens C. A. Mey.	SalInc	Around the road of Yazd-Ardakan	54° 21′	31° 54′	1160
**21**		kali L.	SalKal	Often as weed, generally on soils displaced in different areas of Yazd	54° 20′	31° 53′	300
**22**		Kerneri (Wol.) Osczak. Botsch.	SalKer	Desert areas around Nodooshan	53° 54′	32° 9′	904
**23**		Praoecox Litw.	SalPra	Sand dunes around Ardakan	54° 1′	32° 19′	929
**24**		tomentosa (Moq.) Spach.	SalTom	Across the Yazd desert areas	54° 22′	31° 52′	969
**25**		turcomamca (Litv.) Freitag.	SalTur	Ardakan Around	54° 1′	32° 16′	979
**26**		yazdiana Assadi	SalYaz	Around Kharanagh and Ardakan, Zarrin Rig (endemic of Iran)	54° 40′	32° 21′	1049
**27**	Seidlitzia	cinerea (Moq.) Bge $ Botsch.	SeiCin	Eastern areas of Yazd	54° 21′	31° 53′	550
**28**		rosmarinus (Ehrenbh.) Bge. Ex Boiss.	SeiRos	Beside Yazd-Ardakan Road, Abarkuh desert	53° 16′	31° 8′	1000
**29**	Suaeda	cuminate (C. A. Mey.) Moq.	SuaAcu	Around Ardakan	54° 3′	32° 19′	1171
**30**		aegyptiaca (Hasselq.) Zohary	SuaAeg	Around Ardakan	54° 3′	32° 20′	734

**Fig. 1:**
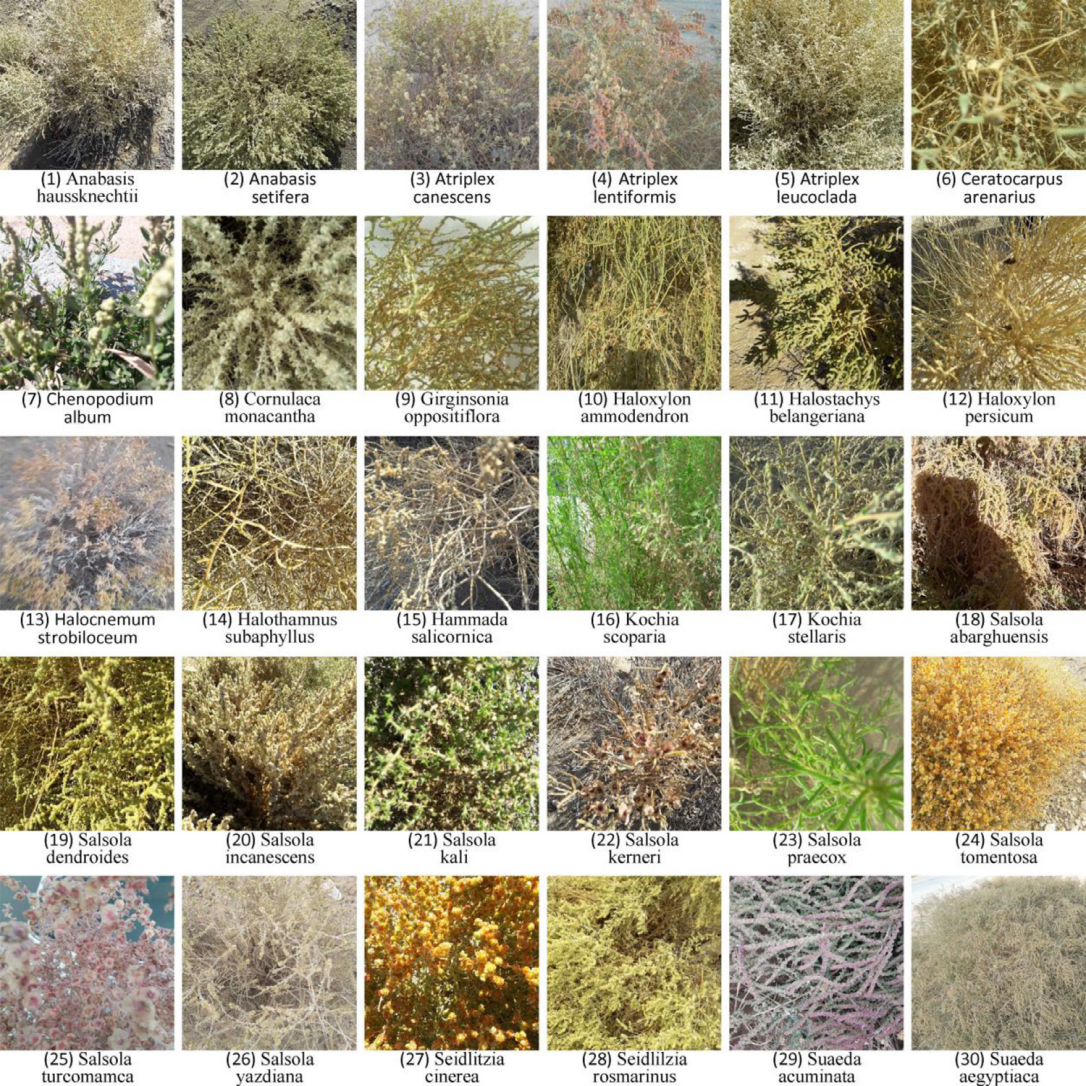
One sample image from each *Chenopodiaceae* species included in the ACHENY dataset.

One sample image from each *Chenopodiaceae* species included in the ACHENY dataset along with its scientific name was shown in Fig. 1.

1. ACHENY dataset classification

**Fig. 2:**
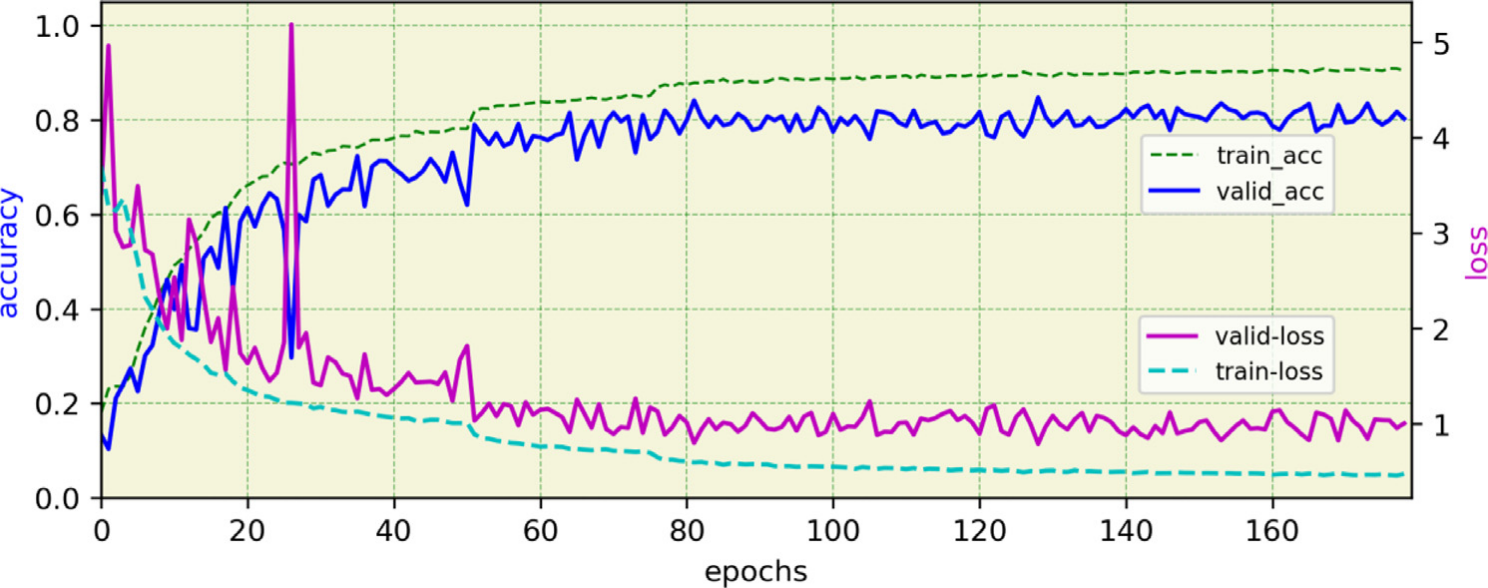
Accuracy and loss time series diagrams based on training epochs related to the Cheno-scratch model.

**Fig. 3:**
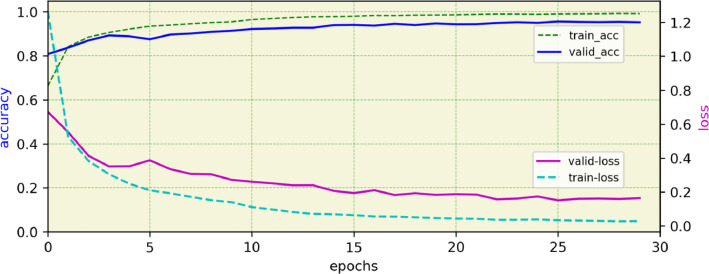
Accuracy and loss time series diagrams based on training epochs related to the Efficient-ACHENY model.

The efficiency of ACHENY dataset is investigated by deep learning models. Hence, we propose two different deep learning models to categorize the ACHENY dataset. First, agile nine-layer, convolutional neural network model, which is called Cheno-scratch. This small and lightweight deep model is trained from scratch. In Cheno-scratch architecture, the size of the input image is designed to be 64 × 64 × 3 to reduce computation and make the model faster. Second, a model is obtained from EfficientNet-B1 [4] by fine-tuning, which is previously trained on ImageNet [7], and is named Efficient-ACHENY. Google's EfficientNet obtain a model by compound scaling up a baseline model [8]. In Efficient-ACHENY the model's width and depth are scaled up according to the associated input size (224 × 224 × 3) which leads to a high-performing model but it increases computational complexity. The visualization of accuracy and loss time series diagrams based on training epochs are shown in Figs. 2 and 3. The details and hyper-parameters of both proposed models are fully described in the related research paper [1]. The experimental results show that both proposed models can perform Chenopodiaceae species recognition with promising accuracy on ACHENY dataset.

## 2 Experimental Design, Materials and Methods

1. Camera specification and setting

Imaging is performed using two different cameras:

  (a) Nikon COOLPIX S2800 digital camera with a 1: 1 (14.9-megapixel) 3864-by-3864 resolution. (b) Samsung SM-J701F mobile with a 1: 1 (3.7-megapixel) 1920-by-1920 resolution.

Both cameras were utilized for image collection in natural light during days.

2. Imaging time and conditions

Studied *Chenopodiaceae* species often have flowers and fruits in the autumn, hence imaging is performed in November and December in their habitat. Outdoors and nature have many uncontrollable factors affecting images, such as light intensity throughout the day, wind blowing, cloudy skies or sunshine, atmospheric precipitation, foggy air, and so on. Imaging was performed at different times of sunny, cloudy and windy days in natural sunlight. Some other factors also affect acquired images, such as camera-to-target distances, viewing angles, location of light sources, and so on.

3. ACHENY dataset in a repository

The ACHENY dataset is available online at Mendeley repository. It is structured in two main folders (ACHENY_size224 and ACHENY_size64), each main folder contains all species images in three zipped files: test.zip contains test images, train.zip contains training images, and validation.zip contains validation images. In each of these zipped files, there are 30 subfolders that were named class names, each contains images in that class. The ACHENY specification table and figure of sample images are also included in main folder.

## Ethics Statement

The work involved neither the use of human subjects nor animal experiments. Data were not collected from social media platforms.

## CRediT authorship contribution statement

**Ahmad Heidary-Sharifabad:** Conceptualization, Methodology, Software, Writing – review & editing. **Mohsen Sardari Zarchi:** Supervision, Writing – original draft. **Sima Emadi:** Validation, Investigation. **Gholamreza Zarei:** Visualization, Data curation, Resources.

## Declaration of Competing Interest

The authors declare that they have no known competing financial interests or personal relationships which have, or could be perceived to have, influenced the work reported in this article.

## Appendix Supplementary materials

Image, application 1
